# Phylogeographic Pattern of the Striped Snakehead, *Channa striata* in Sundaland: Ancient River Connectivity, Geographical and Anthropogenic Singnatures

**DOI:** 10.1371/journal.pone.0052089

**Published:** 2012-12-20

**Authors:** Min Pau Tan, Amirul Firdaus Jamaluddin Jamsari, Mohd Nor Siti Azizah

**Affiliations:** 1 School of Biological Sciences, Universiti Sains Malaysia, Minden, Penang, Malaysia; 2 Centre for Marine and Coastal Studies, Universiti Sains Malaysia, Minden, Penang, Malaysia; University of Lausanne, Switzerland

## Abstract

A phylogeographic study of an economically important freshwater fish, the striped snakehead, *Channa striata* in Sundaland was carried out using data from mtDNA ND5 gene target to elucidate genetic patterning. Templates obtained from a total of 280 individuals representing 24 sampling sites revealed 27 putative haplotypes. Three distinct genetic lineages were apparent; 1)northwest Peninsular Malaysia, 2)southern Peninsular, east Peninsular, Sumatra and SW (western Sarawak) and 3) central west Peninsular and Malaysian Borneo (except SW). Genetic structuring between lineages showed a significant signature of natural geographical barriers that have been acting as effective dividers between these populations. However, genetic propinquity between the SW and southern Peninsular and east Peninsular Malaysia populations was taken as evidence of ancient river connectivity between these regions during the Pleistocene epoch. Alternatively, close genetic relationship between central west Peninsular Malaysia and Malaysian Borneo populations implied anthropogenic activities. Further, haplotype sharing between the east Peninsular Malaysia and Sumatra populations revealed extraordinary migration ability of *C. striata* (>500 km) through ancient connectivity. These results provide interesting insights into the historical and contemporary landscape arrangement in shaping genetic patterns of freshwater species in Sundaland.

## Introduction

**Figure 1:**
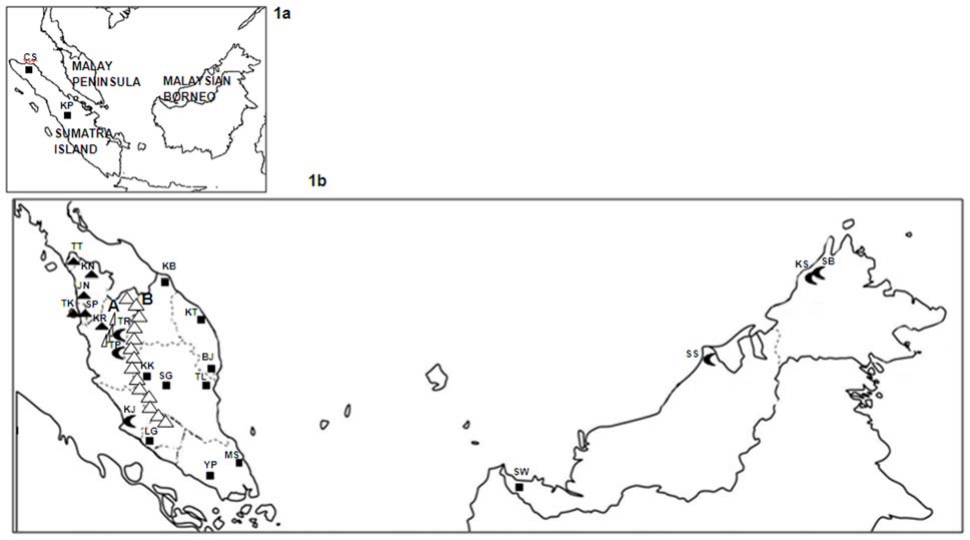
*Channa striata* locality distribution. 1a) Map showing isolated regions involved in this study. 1b) Clustering of *C. striata* populations based on SAMOVA analysis indicated by different symbols: group 1 (triangles), group 2 (new moon) and group 3 (square). A indicates Bintang Mountain Range, B indicates Titiwangsa Montain Ranges. Population abbr.: TT-Timah Tasoh KN-Kuala Nerang JN-Jeniang SP-Seberang Prai TK-Teluk Kumbar KR-Kerian TR-Tanjung Rambutan TP-Tapah KJ-Kajang LG-Linggi YP-Yong Peng MS-Mersing KB-Kota Bahru BJ-Binjai KT-Kubang Bujuk KK-Kuala Krau SG-Sega TL-Tanjung Lumpur SB-Kota Belud KS-Kampung Kesapang SS-Sungai Sibuti SW-Serian CS-Takengon KP-Kampar.

Genetic patterning of ichthyofauna is greatly influenced by ecological processes, anthropogenic factors [1] and geological history [2]. Therefore, understanding palaeogeographical arrangement is as essential as evaluating its contemporary genetic differentiation. During the last glacial maximum, Sumatra Island, Malay Peninsula and Malaysian Borneo (comprising of Sarawak and Sabah), Figure 1a, were bridged by the exposed lowland known as the Sunda shelf. However, melting of the ice sheet during the late glacial period submerged and covered large portions of the Sunda shelf leaving disconnected islands [3] which remain up to the present day. Postglacial invasion of sea water formed the Malay Peninsula isolating it from the Sumatra on the west by the Straits of Malacca and from Borneo on the east by the South China Sea. As a consequence, the subsequent distribution and colonization of the freshwater ichthyofauna was greatly affected as the obligate freshwater taxa found the sea water an insurmountable barrier to dispersion due to the disjunction of the geographic areas [4]. Although the regional separation events are relatively recent (approximately 10,000 years ago), the various large and isolated patches of habitats allowed for independent evolution of surviving individuals along different paths from one another. Thus, investigations of contemporary spatial genetic structuring among these isolated groups could provide insights into the ecological processes and demographic causes of the phylogeographic structuring.

The striped snakehead, *C. striata* (Channidae) is native to, and found naturally throughout freshwater sources across many of Southeast Asian countries and is the most widely distributed species among the snakehead members [5], [6]. Within its native range, it is economically very important, both in the culture and capture sectors [6]. Due to its high value as a food fish, it has been extensively introduced outside of its native range [7] for aquaculture purposes. However, in certain instances introduction has been unintentional and due to the carnivorous and aggressive behavior of this species, it may have serious impact on endemic species, if not well managed. In Malaysia, *C. striata* is often known as haruan or ruan (and several other local names). This species is now commonly found in many freshwater habitats. Its wide occurrence in natural waters coupled by its well-known nutraceutical and pharmaceutical properties, has made it one of the most popular protein food sources, especially among the rural communities [8], [9].

The ability of *C. striata* to colonize natural and artificial reservoirs [10] correlated with its extensive distribution across wide range of natural environmental conditions, suggests that the genetic variation in the wild population could be high. However, how this variation may be geographically distributed, is currently unknown. Indeed, very limited genetic information is known to date for this species. A previous study on genetic structuring and differentiation of *C. striata* in Malaysia using RAPD markers [11] revealed highly significant genetic structuring between the eastern and western divide of Peninsular Malaysia, but contains no record for populations from Malaysian Borneo. The genetic separation in Peninsular Malaysia was effectively marked by the main mountain range that acts as a natural divider, and this finding is in accord with reports on investigations of other local freshwater biota [12]–[14]. Hence the current study is a timely attempt to document molecular data on this channid species in the wider Sundaland region.

Matrilineal markers are potent tools for assessing genetic relationships among individuals within species due to their rapid mutation rates and consequent high genetic variability [15], [16]. Multiple sequences from maternal inherited loci can serve as an informative tool that may reveal demographic changes and genealogical history from millions of years ago by tracing their ancestry back hundreds of generations [17]. The same data could also serve as a guideline for future selection of broodstock by the aquaculture sector and population management [18], [19]. To date, mitochondrial DNA genes have been widely utilised as genetic markers to study the relationships of present day biological populations and to reveal their historical lineages as well as seeking evidence for the existence of ancient biogeographic barriers [20]–[22]. However, they have several disadvantages in population and phylogeographic studies; they can potentially introgress between species [23], [24], are prone to selective sweeps that lead to the loss of mitochondrial diversity within populations [24]–[26] and symbiont-driven changes in mtDNA variation over space specifically in arthropods [27]. Therefore, such investigations must be carefully designed, incorporating supporting evidence from nuclear markers in order to obtain a more robust indication.

The present study was focused on the Malaysian populations from both Peninsular Malaysia and Malaysian Borneo. Two populations from Sumatra, Indonesia were also included. These areas represent a major part of the Sundaland biogeographical region. The NADH dehydrogenase subunit 5 (ND5) gene was utilized as an analytical target. The specific objectives of this study were to characterize genetic diversity at each sampling locality and phylogeographic structuring across the region with respect to natural physical barriers. The initial hypothesis was that adjacent populations share common haplotypes, but even close populations may be significantly structured according to prominent geographical barriers.

## Materials and Methods

### Ethics Statement

Live specimens were collected from local fishermen and wet markets. Sample locations were determined by interview and confirmed to have originated from a single source prior to collection. Clips were taken from the dorsal or caudal fin rays (approximately 0.2 cm x 2 cm) and preserved in 95% ethanol and stored at room temperature (∼25°C) until use. The fish were then returned to the dealers or brought back to the Aquatic Research Centre at Universiti Sains Malaysia (USM), Penang for further research. This study has been approved by the USM Ethics Committee. All practical steps to ameliorate suffering by specimens were taken throughout this study.

### Sampling Location and Collection

Random samples of individuals from a total of 22 wild *C. striata* populations were collected throughout its distribution in Peninsular Malaysia and Malaysian Borneo (Sarawak and Sabah) between 2007 and 2010. Populations were provisionally divided into five regions, northwest Peninsular, central west Peninsular, east Peninsular, southern Peninsular and Malaysian Borneo comprising of Sabah and Sarawak (Table 1). With regards to Peninsular Malaysia, the northwest was defined as areas in west Peninsular confined by the Titiwangsa Mountain Range in the east and Bintang Mountain Range in the south (Figure 1b). Populations to south of the Bintang Mountain Range and up to the southernmost tip of the Titiwangsa Mountain Range were categorised as central west Peninsular. Those east of the Titiwangsa Mountain Range were classified as east Peninsular, while those populations situated at the south without confinement of the Titiwangsa Mountain Range were labeled as southern Peninsular. All populations sampled from the Malaysian Borneo states of Sarawak and Sabah were classified as Malaysian Borneo. Two populations from Sumatra, Indonesia were also included.

**Table 1:** Geographical coordinates of 24 sampled locations.

Region	Population	Latitude (North)	Longitude (East)	N
Northwest Peninsular	1) Timah Tasoh (TT), Perlis	6°35′08″	100°13′14″	6
	2) Kuala Nerang (KN) Kedah	6°14′38″	100°36′22″	14
	3) Jeniang (JN), Kedah	5°48′39″	100°37′27″	6
	4) Seberang Prai (SP), P. Pinang	5°22′08″	100°23′03″	13
	5) Teluk Kumbar (TK), P. Pinang	5°17′04″	100°14′27″	15
	6) Kerian (KR), Perak	4°59′22″	100°32′49″	16
Central west Peninsular	7) Tanjung Rambutan (TR), Perak	4°40′23″	101°08′52″	19
	8) Tapah (TP), Perak	4°11′50″	101°15′48″	10
	9) Kajang (KJ), K. Lumpur	2°59′42″	101°47′51″	14
Southern Peninsular	10) Linggi (LG), N. Sembilan	2°35′07″	102°02′15″	7
	11) Yong Peng (YP), Johor	2°14′39″	103°02′28″	10
	12) Mersing (MS), Johor	2°30′21″	103°49′06″	13
East Peninsular	13) Kota Bahru (KB), Kelantan	6°07′05″	102°14′23″	17
	14) Binjai (BJ), Terengganu	4°13′43″	103°22′03″	10
	15) Kubang Bujuk, Marang (KT), Terengganu	5°16′38″	103°02′55″	16
	16) Kuala Krau, Mentakap (KK), Pahang	3°37′17″	102°23′07″	14
	17) Sega, Raub (SG), Pahang	4°00′58″	101°53′55″	7
	18) Tanjung Lumpur, Kuantan (TL), Pahang	3°47′46″	103°20′09″	7
Malaysian Borneo	19) Kota Belud (SB), Sabah	6°21′03″	116°25′58″	14
	20) Kampung Kesapang (KS), Sabah	6°21′55″	116°26′50″	14
	21) Sungai Sibuti, Miri (SS), Sarawak	4°00′43″	113°46′31″	12
	22) Serian (SW), Sarawak	1°02′43″	110°45′03″	15
Sumatra	23) Takengon (CS), Aceh	4°36′53″	96°50′45″	6
	24) Kampar (KP), Riau	0°18′12″	101°22′01″	5
Total				280

Number of individuals per site (N).

### Mitochondrial DNA Extraction and Analysis

DNA templates were isolated using AquaGenomic™ DNA isolation kits (MultiTarget Pharmaceuticals, Salt Lake City, Utah 84116) following the manufacturer’s protocol. Aliquots of purified DNA isolates were used as templates for PCR amplification of the complete ND5 gene. The primer pair L12321-Leu (5′- GGTCTTAGGAACCCAAAACTCTTGCTGCAA -3′) and H13396-ND5 (5′- CCTATTTTKCGGATGTCYTG-3′) [12] were used. The PCR mixture contained 50–100 ng of genomic DNA, 0.05 µM of each primer, 0.17 mM of dNTP, 1.4×PCR buffer, 1 mM MgCl_2_and 1.67 U of *Taq* polymerase (all from iNtRON). The PCR was conducted in 30 µl total reaction volume in an MJ PTC-200 Thermal Cycler (MJ Research, Waltham, MA, USA). Amplification conditions were: initial incubation at 94°C (2 min); 35 cycles at 94°C (20 sec), 55°C (20 sec), 72°C (1 min 10 sec), final extension at 72°C (5 min) and a final hold at 10°C. The PCR products were visualized on a 1.7% agarose gel and stained with ethidium bromide to confirm successful amplification. The PCR product was purified using QIAGEN purification kits (QIAGEN Sciences, Maryland 20874, USA) according to the manufacturer’s instruction. At the final step, 30 µl product was eluted and 5 µl of the total elution was used to assess the quality of purified product on a 1.7% agarose gel. All purified products were sent for DNA sequencing (First BASE Laboratories Sdn Bhd, Selangor, Malaysia) and reading from both DNA strands. Multiple sequences were aligned and all unambiguous operational taxonomic units (OTUs) were compiled and edited using ClustalW implemented in MEGA 4.0 [28]. All haplotype sequences have been submitted to GenBank under accession numbers HQ384453-HQ384478 and HQ438583. DNA sequences were translated into protein to ensure accurate alignment and detection of numts, if present. The aligned sequences were then exported to Collapse 1.2 [29] to construct a haplotype datasheet. Haplotype distribution among all populations was summarized manually from DnaSP programme output [30]. Then, the intra- and inter-population variation patterns of these haplotypes were analyzed. The complete aligned dataset was analyzed for variable nucleotide sites, parsimony informative sites, number of haplotypes, synonymous and non-synonymous amino acid substitutions and nucleotide frequencies in MEGA 4.0. Haplotype/gene diversity (H_d_) and nucleotide diversity (π) were calculated to describe DNA polymorphism at each sampling site using Arlequin 3.1 [31].

### Evolutionary Relationships Among Haplotypes

Gene trees were constructed using Neighbor-Joining (NJ) [32] and Bayesian phylogenetic tree building methods in MEGA 4.0 [28] and BEAST v1.7.1 [33], respectively. GenBank sequences of *C. micropeltes* (HQ 438584) and *C. argus* (10251173: [34]) were included as outgroups. Kimura 2-Parameter [35] evolutionary distances were used with the NJ method and the confidence levels at each node assessed by 1000 bootstrap replications [36]. Bayesian inference of phylogeny was implemented in BEAST and data files were compiled using the BEAUTi routine in the BEAST package with the following parameters: General Time Reversible nucleotide substitution model incorporating Gamma site heterogeneity model (GTR+G), a relaxed molecular clock with uncorrelated lognormal distribution [37], randomly generated starting trees with tree prior coalescent-constant size [38]. GTR+G substitution model was selected for it is an independent, finite site and generalized time reversible model while gamma allows substitution rate variation among sites in the data [33]. Age calibration or time divergence analysis was not considered in this study as it is not our prior focus and thus only gene tree with posterior probability results were discussed. The analysis incorporated 10,000,000 generations with parameters logged every 1000 generations. This analysis was run three times and the log output files were combined using LogCombiner (in BEAST package). The software package Tracer v1.4 [39] was used to visualize the performance of the analysis by checking the Effective Sample Size (ESS) values. The final target tree was viewed in FigTree [40] after summarization from a sample of trees produced in BEAST by using TreeAnnotator (in BEAST package). To view the evolutionary relationships among haplotypes, a phylogenetic network of all haplotypes was constructed by median joining calculation in Network 4.6 [41].

### Defining Groups of Populations, Genetic Differentiation and Gene Flow Estimates

A spatial analysis of molecular variance was conducted using SAMOVA v.1.0 [42] to identify genetically similar groups of populations and to evaluate the amount of genetic variation among the partitions. The optimal number of groups (*k*) was determined based on the highest value of variance among groups (F_CT_), incorporating information on haplotype divergence and geographical proximity. Subsequently, a hierarchical analysis of molecular variance (AMOVA) was conducted to infer the relative contribution to variance among groups (F_CT_), among populations within groups (F_SC_) and within populations. Based on the SAMOVA population structure estimate (*k*), the population pairwise comparison statistic, F_ST_ that calculates relative genetic differentiation between populations was determined in Arlequin 3.1 to evaluate the significance or otherwise of differences among populations and spatial population structuring. The analysis used Kimura 2-Parameter data and statistically significant pairwise comparisons were tested with 10,000 permutations. Significant probability values were adjusted by performing the False Discovery Rate Procedure (FDR) at α = 0.05 which controls the family wise error rate (FWER), a conservative type I error rate that originates from multiplicity [43]. Haplotype-based statistics (H_ST_) and sequence-based statistics (N_ST_ [44] and K_ST_*) were also employed using 1000 permutations [45] in DnaSP programme [30] as additional measures of genetic differentiation. Using the same programme, gene flow estimates (Nm) based on both haplotype-based and sequence-based statistics were derived as in [46] and [45], respectively. Genetic distances between populations were calculated based on Kimura 2-Parameter distance method as implemented in MEGA 4.0.

## Results

A total of 280 individuals from 24 populations were successfully PCR amplified for mtDNA sequence variation in the ND 5 gene. A final truncated target length of 1017 bp was obtained after alignment and editing of ambiguous sequences. Sample size varied from five to 19 individuals per population with an average of 12. The final alignment of sequences revealed a total of 53 segregating sites (54 mutation sites and 22 parsimony informative sites) defining 27 putative haplotypes with 14 (51.9%) of them being private haplotypes (Table S1). The average nucleotide composition was 28.4% A, 27.6% T, 30.3% C and 13.7% G. Nucleotide substitution rate was approximately 1∶3.9 transversion to transition, occurring with a ratio of 2.3∶1:14.3 at codon position 1, 2 and 3 respectively. Out of a total of 339 amino acid sequences, nine amino acid mutations occurred as a result of nucleotide substitutions at the various codon positions (5 at the first codon position, 3 at the second and 1 at the third). This is typical of ND5 gene that is known to accumulate more informative protein variation at the first and second codon positions [47]. As an illustration, Hap25 from collection location LG had 18 polymorphic sites (see Table S1) which resulted in two amino acid substitutions.

Closer observation revealed that these amino acid substitutions were mainly regional specific, if not population specific. Thus, Hap01, Hap05, Hap19, Hap21 and Hap23 were specific to the six northwest Peninsular populations (TT, JN, KN, SP, TK and KR) (Table S1 & S2). In contrast, *C. striata* from all other regions (central west Peninsular, east Peninsular and southern Peninsular, Sumatra and Malaysian Borneo) shared other common haplotypes – Hap03, Hap04, Hap07, Hap11, Hap12, Hap13, Hap14 and Hap15. However, nucleotide substitution at several sites (e.g. in Hap02, Hap09, Hap10, Hap16, Hap17 and Hap18) further differentiated the central west Peninsular and Malaysian Borneo (except SW) from other populations.

Nevertheless, several unexpected findings were made, for example the central west Peninsular Malaysia populations had haplotypes in common with the Malaysian Borneo populations. The dominant haplotype of Malaysian Borneo populations (SS, SB and KS) was Hap02 and this was also found in 28.6% of individuals in one of the central west Peninsular Malaysia population, KJ and in single individuals from KR and KK. Moreover, a single individual from KS population (Malaysian Borneo) was also found to share Hap09 with another two central west Peninsular Malaysia populations, TR and KJ.

### Genetic Diversity within Population

Overall haplotype diversity was 0.9±0.006. In general, genetic diversity was the highest in central west Peninsular. Here, TR was most polymorphic with 20 variable sites, three being singletons (Table 2). This was followed by KJ (16), KK (14) and SG (12) polymorphic sites. Number of haplotypes in each population ranged from one to eight with a mean of 2.42 per population where TR possessed the highest number of haplotypes while nine populations showed a total absence of genetic variation. Hence, a wide range of within population genetic variability was observed (H_d_  =  0.00 to 84.62% and π  =  0.00 to 0.65%). In common with findings made using other variability measures, nucleotide diversity was highest in SG (0.65%), followed by KJ (0.58%), TR (0.57%), JN and KK (0.39%).

**Table 2:** Summary of genetic diversity of individual population.

				Genetic diversity		
No	Pop	N	#V	H	H_d_	Π
1	TT	6	0	1	0	0
2	KN	14	1	2	0.143	0.0001
3	JN	6	10	3	0.600	0.0039
4	TK	15	0	1	0	0
5	SP	13	1	2	0.154	0.0002
6	KR	16	10	4	0.350	0.0018
7	TR	19	20	8	0.830	0.0057
8	TP	10	0	1	0	0
9	KJ	14	16	6	0.846	0.0058
10	LG	7	7	4	0.600	0.0025
11	YP	10	0	1	0	0
12	MS	13	2	2	0.539	0.0011
13	BJ	10	0	1	0	0
14	KB	17	8	3	0.228	0.0010
15	TL	7	2	2	0.476	0.0009
16	SG	7	12	3	0.762	0.0065
17	KT	16	0	1	0	0
18	KK	14	14	5	0.593	0.0038
19	SW	15	0	1	0	0
20	SS	12	9	2	0.303	0.0027
21	SB	14	0	1	0	0
22	KS	14	3	2	0.143	0.0004
23	CS	6	1	2	0.600	0.0006
24	KP	5	0	1	0	0
Group 1		70	15	9	0.537	0.0012
Group 2		83	25	12	0.708	0.0037
Group 3		127	44	15	0.817	0.0031

Sample size (N), number of variable sites (#V), number of haplotypes (H), haplotype diversity (H_d_) and nucleotide diversity (π).

### Evolutionary Relationships among Haplotypes

**Figure 2:**
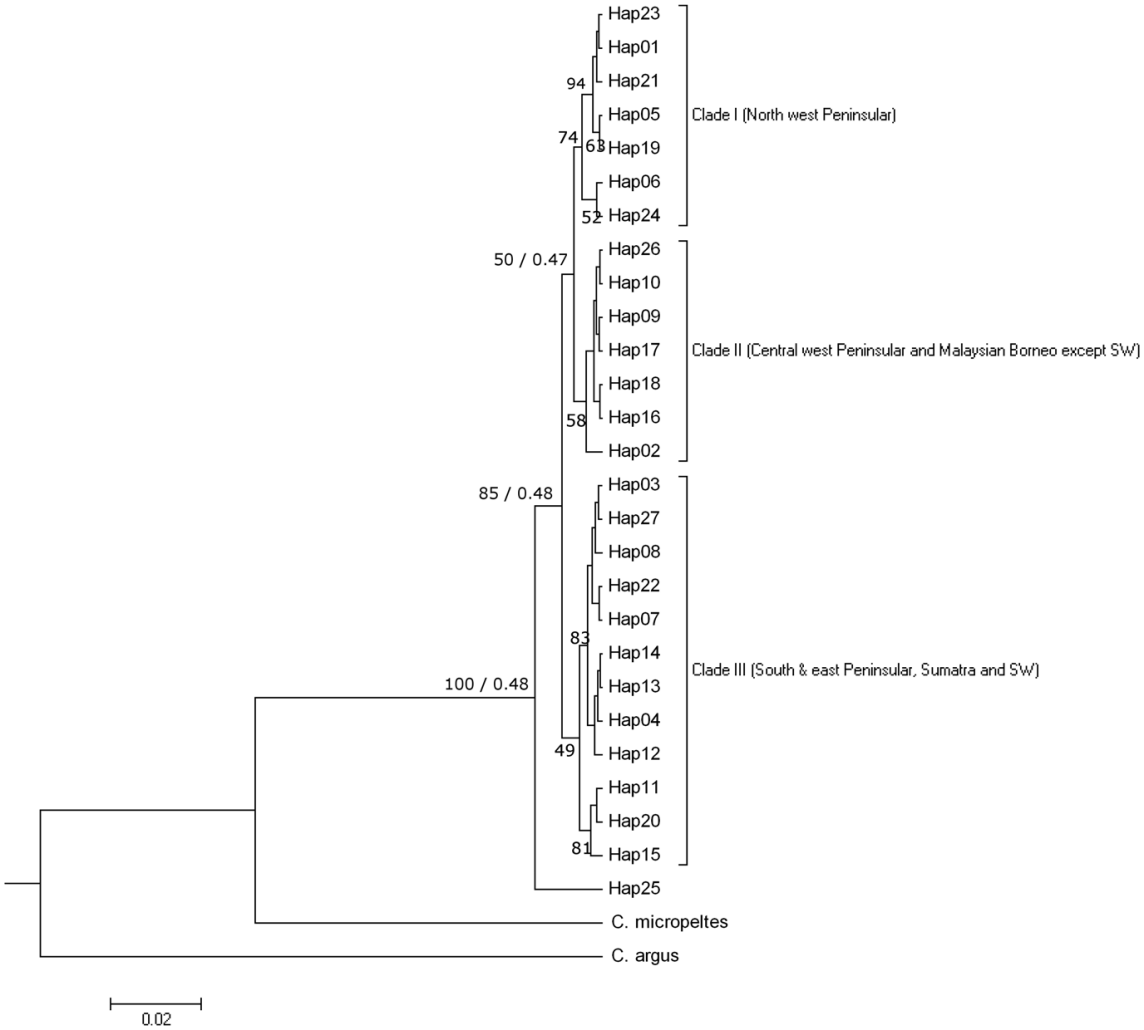
Bayesian clustering tree inferred from ND5 gene. Selected NJ bootstrap/Bayesian posterior probability values denoted at branch/node for better visualization.

Gene trees inferred by NJ and Bayesian tree clustering methods recovered the same topology. All 27 haplotypes could be assigned to three major lineages marked by more or less robust bootstrap values (>70%) as shown in the NJ tree (Figure 2). However, relatively low bootstrap values/posterior probabilities were recovered for Clade II and III (58 and 49%, respectively) while the internal node of Clade III showed bootstrap values higher than 80%. Clade I consists of haplotypes from northwest Peninsular Malaysia and is the sister taxon to Clade II that clustered haplotypes in populations from central west Peninsular and Malaysian Borneo (except SW). Clade III consists of haplotypes mainly from east Peninsular, southern Peninsular, Sumatra and SW. The genetically distant haplotype, Hap25, from the LG population forms the basal group in the NJ tree.

**Figure 3:**
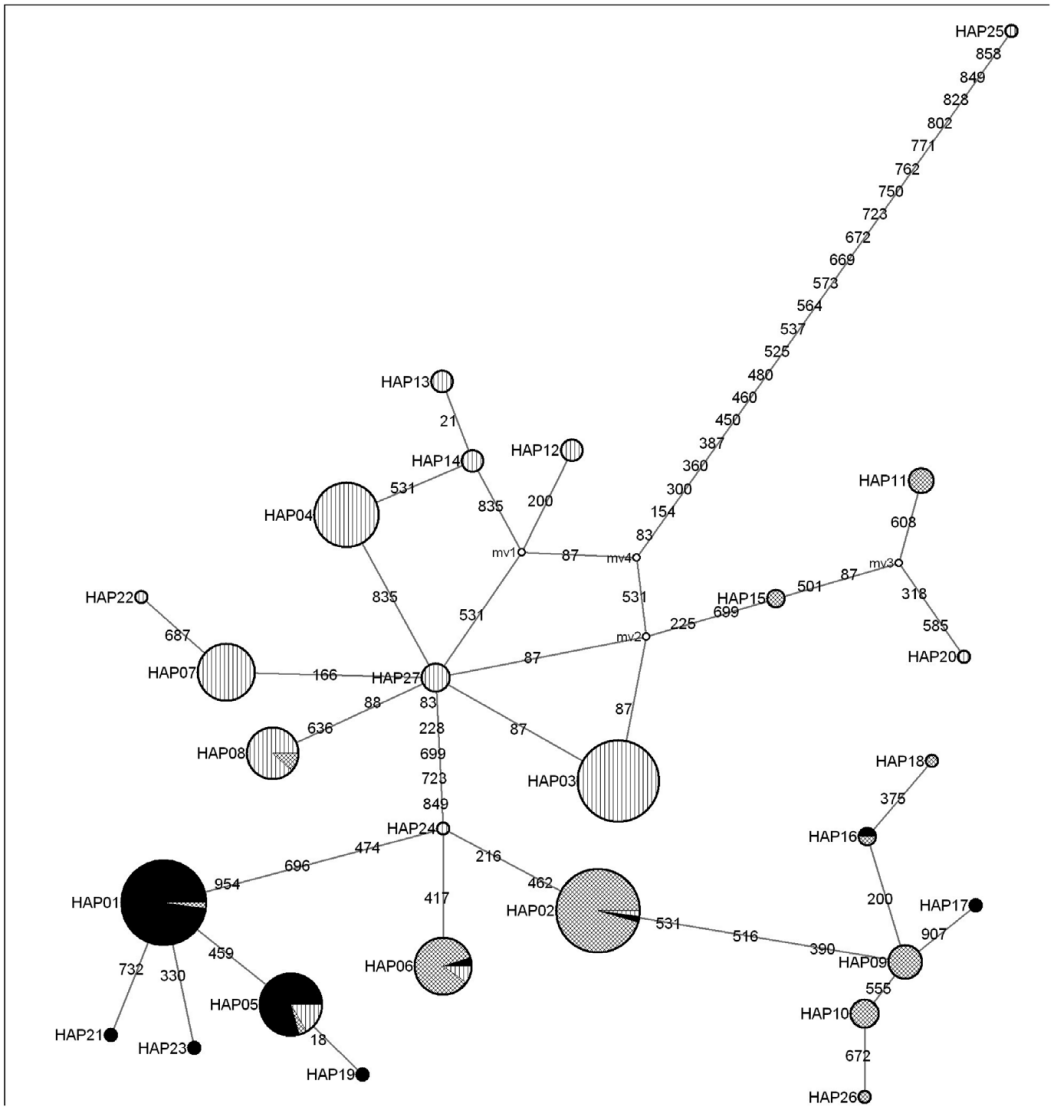
Haplotype network diagram inferred from mtDNA ND5 gene. Solid pattern represents populations assigned to clade I (northwest Peninsular), diagonal cross pattern to clade II (central west Peninsular and Malaysian Borneo except SW) and vertical pattern to clade III (east Peninsular, southern Peninsular, Sumatra and SW). mv = median vector. Numbers in bold are nucleotide mutation sites.

The haplotype network diagram (Figure 3) illustrated that Hap01, Hap02 and Hap03 were the dominant haplotypes in Group 1. northwest Peninsular, Group 2. central west Peninsular and Malaysian Borneo (except SW) and Group 3. east Peninsular, southern Peninsular, Sumatra and SW, respectively. The ubiquitous haplotypes (Hap02, 05 and 06) were shared by all three groups while another three (Hap01, 08 and 16) were shared between any two of the three groups. Homoplasy, as indicated by multiple substitutions of nucleotide at a single site [48] was detected between Hap03 (east Peninsular) and Hap27 (Sumatra).

Interestingly, there were some slight discrepancies as observed in the assignation of Hap11 and 15 using gene tree and network diagram. In the gene tree, they were grouped closely to Clade III while they seemed to be closer to Clade II in the haplotype network diagram.

### Defining Groups of Populations, Genetic Differentiation and Gene Flow Estimates

In the SAMOVA analysis, increasing the number of *k* clusters from 2<*k*<20 directly increased the values of variance among groups (F_CT_) suggesting that the populations were highly structured. Therefore, *k* cluster grouping based on the highest F_CT_ was not very useful to assess population relationships. Thus, based on the phylogenetic NJ analysis, *k* clusters value was set at 3 (Figure 2, Table 3). Genetic variation was largely distributed among groups (F_CT_ = 64.59%), followed by within populations (19.95%) and finally between populations within groups (F_SC_ = 15.45%). The grouping obtained was precisely the same as that defined by the previous phylogenetic analysis.

**Table 3:** SAMOVA analysis on *C. striata* populations inferred from mtDNA ND5 gene.

Group 1	Group 2	Group 3
Timah Tasoh	Tanjung Rambutan	Linggi
Kuala Nerang	Tapah	Yong Peng
Jeniang	Kajang	Mersing
Teluk Kumbar	Sungai Sibuti	Binjai
Seberang Prai	Kota Belud	Kota Bahru
Kerian	Kampung Kesapang	Tanjung Lumpur
		Kubang Bujuk
		Kuala Krau
		Sega
		Takengon
		Kampar
		Serian

Based on pairwise F_ST_ values, 89.5% of the comparisons showed significant population differentiation (p<0.05) after FDR adjustment (table not shown). Out of 276 possible comparisons, three of the pairwise estimates were negative. Adjacent populations in the same group (as defined in SAMOVA- table 3) generally had closer relationships with each other (even though the population pairs were themselves fairly well differentiated compared with those between groups. Interestingly, however, pairwise F_ST_ revealed that the KJ (central west Peninsular) population was closely related to the Malaysian Borneo population, SS, while the KP population (Sumatra) was not differentiated from SG (east Peninsular) and nor from two populations in southern Peninsular, LG and MS. These observations were in agreement with the gene trees which divided the populations we investigated into the three clades.

Genetic differentiation estimates for all populations based on haplotype-based statistics (H_ST_) and nucleotide sequence-based statistics (N_ST_ and K_ST_*) were high (0.67 to 0.72) and significant (p<0.001), consistent with the F_ST_ statistic results. Gene flow estimates among populations were low (Nm = 0.25 and 0.20, haplotype-based and sequence-based, respectively).

Mean genetic distances between populations computed based on Kimura 2-parameter values ranged from 0.00%–1.20% (table not shown) with an overall mean distance of 0.62%. Comparisons of pairwise F_ST_ between groups showed the highest differentiation between groups 1 and 3 (74.35%) followed by between groups 2 and 3 (58.86%) and the lowest between groups 1 and 2 (57.63%). This pattern was further supported by the divergence analyses. All pairwise comparisons were significantly structured in F_ST_ at 95% confidence level.

## Discussion

### Genetic Diversity

The SG, TR, KJ and LG samples were shown to have come from among the most highly variable populations (Table 2) i.e. those that significantly contributed to the total genetic diversity in *C. striata* populations within the regions studied. Overall, relatively high haplotype diversities were observed in most *C. striata* populations (Table 2), a phenomenon often noted in freshwater fishes inhabiting non-glaciated regions (during the past glaciations era) or temperate regions [49], [50]. In support of this, an earlier study of *C. striata* populations mainly from the Mekong and Chao Phraya rivers found them to exhibit relatively high population haplotype diversity (0.97) inferred from mtDNA cytochrome b gene sequence data [51]. Similar high genetic diversities have been observed in other freshwater fish populations; the cyprinid, *Acrossocheilus paradoxus* (H_d_ = 1.00) inferred from multiple mtDNA segments [52], four Hawaiian freshwater fishes, *Lentipes concolor*, *Stenogobius hawaiiensis*, *Sicyopterus stimpsoni* and *Awaous guamensis* (0.47 to 0.98) inferred from both coding and non-coding regions of mtDNA [53].

In contrast, nine *C. striata* populations showed a total absence of genetic variation, which was not simply related to sample size. Several of the smaller populations (e.g. such as JN, N = 6, H_d_ = 0.6) harbored higher genetic variation than some of the larger ones (N>10, e.g. TK, N = 15) which showed monomorphism or lower haplotypic variability (Table 2). Thus, the observed monomorphism may just be population specific. Being a commercially important fish could lead to a small effective population size as a consequence of one or, more likely, several plausible factors such as overexploitation [55], habitat fragmentation [56] or habitat loss due to environmental perturbation including human activities [57] resulting in genetic bottlenecks that may have led to inbreeding [54].

### Phylogeographic Structure

The mtDNA ND5 gene data defined the populations into three major lineages (Table 3, Figure 1) i.e. 1)northwest Peninsular, 2)central west Peninsular and Malaysian Borneo except SW, and 3)east Peninsular, southern Peninsular, Sumatra and SW. However, the less robust support for Clade II and Clade III could possibly be due to insufficient time for lineage sorting to complete. Hence, a better support for the inferences could be obtained by utilizing multiple genes with different modes of inheritance (58–60). On the other hand, the slight discrepancy seen in the clustering of Hap11 and 15 could be explained based on the SAMOVA analysis. According to the SAMOVA, Hap11 and 15 belonged to populations in Clade II i.e. central west Peninsular Malaysia. Yet, when constructing the gene tree, both Hap11 and 15 seemed to be related closer to Clade III., i.e. southern Peninsular, east Peninsular, Sumatra and SW. This is not surprising as both haplotypes were found in KJ, a population located at the border between Clade II and III. The close genetic relationship could be explained as the result of gene flow between these two clades.

#### Ancient river connectivity

Our study provides yet further evidence of historical events associated with the palaeo North Sunda River system during the Pleistocene era [3] as highlighted by the genetic distinctiveness of the SW population (west Sarawak) from other Malaysian Borneo populations in addition to its genetic proximity with several populations in southern Peninsular, east Peninsular Malaysia and Sumatra (Figure 1). A study of the river catfish, *Hemibagrus nemurus* within the Southeast Asian region has shown a genetically and morphologically characteristic form of *H. nemurus* is found in the Kapuas River (west Borneo) as distinct from other Sarawak populations (Sadong River at Serian and Rajang River at Kapit and Sibu) [61]. This finding was attributed to ancient isolation of these two regions during the Pleistocene Epoch. However, the genetic affinities of our SW *C. striata* population with the southern Peninsular and Sumatra populations suggest ancient drainage connectivity, possibly via the North Sunda River system. This ancient drainage was believed to have connected many rivers of Sumatra and Borneo, and thus having a major influence on freshwater fish dispersal between these two regions [62]. Based on the cytochrome b gene, haplotype sharing pattern noted between *H. macrolepidota* populations from southern Peninsular Malaysia with southern and western Sarawak populations has been taken to reflect recent geographic isolation between the two regions [63]. In another study, the sharing of haplotypes in *Tor tambroides* between Sarawak and Perak (central west Peninsular) populations, inferred from partial COI gene was hypothesized to be a consequence of a historical drainage connection during the last Pleistocene glaciation period [64]. Furthermore, genetic similarity found in samples of the freshwater species, *Barbonymus schwanenfeldii* from Peninsular Malaysia and Sarawak inferred from mitochondrial cytochrome b gene sequence data was in concordance to the separation of the Borneo Island from mainland Peninsular during the late Pleistocene [4]. In our study, when the partition statistics, *k,* was set at a value of 3, the SW population (west Sarawak) showed closest relationship to the populations belonging to east Peninsular, southern Peninsular and Sumatra though they are presently separated by a great expanses of sea namely the South China Sea and Straits of Malacca respectively.

#### Geographical highland barriers to genetic connectivity

Population isolation over a prolonged period of time, most prominently due to geographical barriers may have permitted the development of characteristic mtDNA variants in individual populations in this species. Evolutionary processes occurred independently in the various isolated regions and have now resulted in the clustering of three distinct monophyletic molecular groups. The first group consisted of six Peninsular Malaysia populations situated in northwest Peninsular (TT, KN, JN, SP, TK and KR) where they were physically isolated from populations to the south of them by the Bintang Mountain Range. This mountain acted as an effective barrier to gene flow, which would have constrained short-range migrations between populations separated by this divide.

The other significant structuring was observed between populations from the central west Peninsular and east Peninsular Malaysia (Clade III). The main mountain range (Titiwangsa Mountains) which was formed during the late Triassic (200mya) had acted as a natural divider to terrestrial as well as riverine biota since its formation. A similar pattern of separation has been reported by several researchers investigating freshwater fishes within this region; the freshwater cyprinid, *Labiobarbus leptocheilus* [65]; the marble goby, *Oxyeleotris marmoratus* [12], the river terrapin *Batagur baska* [13] and the climbing perch, *Anabas testudineus* [14]. Not unexpectedly, the three central west Peninsular Malaysia populations; TR, TP and KJ were significantly differentiated from all of the east Peninsular populations (KB, KT, BJ, SG, TL and KK) and of the southern Peninsular populations (LG, YP and MS) too. More interestingly, these same central west Peninsular populations were also closely related to the Malaysian Borneo (except SW); specifically SS, SB and KS (Group 2). However, while ancient connectivity was the likely reason for the close relationships between west Sarawak, east Peninsular, southern Peninsular and Sumatra populations as discussed in the previous section, evidence for such connectivity has never previously been reported between central west Peninsular and Malaysian Borneo (except SW) populations.

#### Human-mediated translocation involving central west Peninsular and Malaysian Borneo (excluding SW) populations

Lack of genetic differentiation between two adjacent populations is not unexpected but when two distantly located populations are found to be homogenous, then historical ecology and demographic explanations may be important factors to consider when trying to understand the lack of genetic patterning. As discussed above, based on similar investigations of other freshwater species in this area several of the unexpected findings of this study may be attributed to ancient connectivity. The native range of *C. striata* as reported by the United State Geological Survey (USGS, 2011) shows that this species is not indigenous to most of the eastern part of Sarawak and the state of Sabah. Thus, in this case data for populations SS, SB and KS strongly suggest human mediated translocation as the reason for the presence of these populations in these regions. However, there has been one report documenting *C. striata* as native to Sabah [62] and which now needs to be further investigated. Nevertheless, the special capabilities of the species to breathe air and stay alive during shipping [7], in addition to its attractive economic properties, has made it a popular species for introduction to other non-native areas. Shipping of live *C. striata* from Borneo to Singapore took place back in the 1950s as well as by unintentional translocation due to human activities in the past [66]. Unfortunately, there is no record of the fish being transported from Peninsular Malaysia to Sabah and Sarawak.However, as yet, there is no wider support for the alternative ancient river hypothesis to explain connectivity between the central west Peninsular and Malaysian Borneo particularly Sabah i.e. evidence for any such historical link is presently absent [3], [4]. There is some circumstantial support; the reduced genetic diversity observed at these sites compared with its genetically close relatives in central west Peninsular populations is concordant with a founder effect. The colonization of newly created habitats by a low effective population number of founders is concomitant with a high rate of inbreeding and hence later characterized by a low genetic variation in the introduced population [67], exactly as is observed in the Sabah populations. Furthermore, the haplotypes found in populations of SB and KS (both from Sabah of Malaysian Borneo) were also a subset of the haplotypes found in the KJ population. In short, human translocation whether intentionally or otherwise seemed to be the most plausible explanation for the presence of related haplotypes separated by formidable marine divide.

#### High genetic structuring among *C. striata* populations

A high level of genetic structuring was apparent among the populations of *C. striata* in this study as revealed by haplotype-based (H_ST_) and sequence-based (N_ST_ and K_ST_*) statistics. The population pairwise F_ST_ values are also consistent with the low degree of gene flow estimates between populations (Nm = 0.25 and 0.20 respectively for haplotype-based and sequence-based). High genetic structuring particularly of non-migratory freshwater fishes have been well documented [4], [68], [69]. This is due to restrictive physical barriers separating populations between pairs of adjacent populations, though *C. striata* is well-known to be capable of short distance migration over land [10]. Therefore, when this occurs adjoining populations may effectively become panmictic, sharing or exchanging alleles as observed in this study among populations within regions.

#### The ancient Siam River connectivity

Another interesting finding in this study was the private unique haplotype, Hap27, from the KP population, Sumatra. Despite geographically distant separation, KP was grouped with the SG population (east Peninsular) and LG and MS (southern Peninsular); see Figure 2 and due to similarities between the sequences of Hap 27 and Hap 03. This is another probable indication of connectivity between Sumatra and east Peninsular Malaysia during the last glacial period when the sea level was lower than the present day. The existence of an ancient Siam River system has been hypothesized as “a large river system that included Sumatra’s Kampar River that ran through Straits of Singapore… likely joined branches from the Gulf of Thailand… and must have included major contributions from Endau River, Pahang River, Terengganu River and Kelantan River of the east coast of Peninsular Malaysia” [3]. When the interconnecting river system between the Sumatra and Peninsular Malaysia was submerged by rising sea level, lateral escapes and recolonization inwards into east Peninsular Malaysia occurred as revealed by the present study.

In support of this account, another population from Sumatra, CS was also found to be clustered in the same group, further illustrating the close genetic relationship between populations from these two regions. Similar close genetic relationships were also observed between populations of the climbing perch, *Anabas testudineus* from Aceh (Sumatra) and Terengganu (east Peninsular Malaysia) [14]. The genetic proximity of these two regions was suggested as being due to a common origin, most probably through natural migration via the palaeo river system which traversed across the two regions during the most recent Pleistocene glaciations.

During unfavourable periods of marine invasion, local adaption or migration for survival even beyond atypical migratory range (approximately 500 km as measured along the ancient Siam River System) was the only means of escape for this *C. striata* population leading to the pattern of genetic diversification which still persist until today. Though the populations from east Peninsular harboured higher genetic variation, the lower genetic variation detected in the KP population might be due to demographic bottleneck. Further investigation on samples from Sumatra should be undertaken to elucidate the phylogenetic relationships of this group.

### Conclusions

Genetic patterning among populations of the striped snakehead, *C. striata* in the Sundaland was highly influenced by the interplay of several factors. The most prominent finding of this study was the segregation of this obligate freshwater species into three highly structured and significant phylogenetic groups, limited by effective geographical barriers and thus low gene flow between each population and phylogenetic region. Anthropogenic activity may also have played a major part via the probable translocation of this species from central west Peninsular Malaysia to as far away as Malaysian Borneo as detected here using a maternal lineage marker. Furthermore, ancient dispersal through the palaeo river system running across two presently isolated regions was also apparent indicating the typical Pleistocene glacial signature of the genetic structure of freshwater fishes in the Sundaland. This study has indirectly revealed the dispersal power of *C. striata* when dispersal limitation was broken down and its high mobility and rapid adaptability into a newly colonized area.

## Supporting Information

Table S1 **Haplotype frequency and nucleotide polymorphic sites encoded by 27 haplotypes.** Non-synonymous amino acid substitution is indicated by *
(DOCX)

Table S2 **Haplotype distribution across 24 populations.** Abbreviation of population name is as listed in Table 1.
(DOCX)

## References

[1] Lande R (1998) Anthropogenic, ecological and genetic factors in extinction and conservation. Researches on Population Ecology 40(3): 259–269.

[2] Turner TF, McPhee MV, Campbell P, Winemiller KO (2004) Phylogeography and intraspecific genetic variation of prochilodontid fishes endemic to rivers of northern South America. Journal of Fish Biology 64: 186–201.

[3] Voris HK (2000) Map of Pleistocene sea levels in Southeast Asia: shorelines, river systems and time duration. Journal of Biogeography 27: 1153–1167.

[4] Kamarudin KR, Esa Y (2009) Phylogeny and phylogeography of *Barbonymus schwanenfeldii* (Cyprinidae) from Malaysia inferred using partial cytochrome b mtDNA gene. Journal of Tropical Biology and Conservation 5: 1–13.

[5] Mohsin AKM, Ambak MA (1983) Freshwater fishes of Peninsular Malaysia. Kuala Lumpur: Penerbitan Universiti Pertanian Malaysia. 284p.

[6] Kottelat M, Whitten AJ, Kartikasari SN, Wirjoatmodjo S (1993) Freshwater Fishes of Western Indonesia and Sulawesi. Hong Kong: Periplus Editions (HK) Ltd. 293p.

[7] Courtenay WR Jr, Williams JD, Britz R, Yamamoto MN, Loiselle PV (2004) Identity of introduced snakeheads (Pisces, Channidae) in Hawai’i and Madagascar, with comments on ecological concerns. Honolulu: BISHOP Museum Press. 13p.

[8] Mat Jais AM, McCulloch R, Croft K (1994) Fatty acid and amino acid composition in haruan as a potential role in wound healing. General Pharmacology 25(5): 947–950. 7835642 10.1016/0306-3623(94)90101-5

[9] Baie SH, Sheikh KA (2000) The wound healing properties of *Channa striatus* cetrimide cream: tensile strength measurement. Journal of Ethnopharmacology 71, n. 1–2: 93–100. 10.1016/s0378-8741(99)00184-1 10904151

[10] Amilhat E, Lorenzen K (2005) Habitat use, migration pattern and population dynamics of chevron snakehead *Channa striata* in a rainfed rice farming landscape. Journal of Fish Biology 67 (Supplement B)23–24.

[11] Ambak MA, Ambok BAM, Ismail P, Bui MT (2006) Genetic variation of snakehead (*Channa striata*) populations using random amplified polymorphic DNA. Biotechnology 5(1): 104–110.

[12] Ruzainah A (2008) Population genetic studies of marble goby *Oxyeleotris marmoratus* (Bleeker, 1852) in Malaysia using microsatellite and mitochondrial DNA markers. PhD thesis, Universiti Sains Malaysia.

[13] NorKarmila D (2009) Morphometric and genetic variability of river terrapin, (*Batagur baska*) and painted terrapin, (*Batagur borneoensis*). MSc thesis, Universiti Sains Malaysia.

[14] Jamsari AFJ, Muchlisin ZA, Musri M, Siti Azizah MN (2010) Remarkably low genetic variation but high population differentiation in the climbing perch, *Anabas testudineus* (Anabantidae), based on mtDNA control region. Genetics and Molecular Research 9(3): 1836–1843. 20845309 10.4238/vol9-3gmr933

[15] Brown WM, George MJ, Wilson AC (1979) Rapid evolution of animal mitochondrial DNA. Proceedings of the National Academy of Sciences of the United States of America 76(4): 1967–1971. 109836 10.1073/pnas.76.4.1967 PMC383514

[16] Jenuth JP, Peterson AC, Fu K, Shoubridge EA (1996) Random genetic drift in the female germline explains the rapid segregation of mammalian mitochondrial DNA. Nature Genetics 14: 146–151. 8841183 10.1038/ng1096-146

[17] Harpending HC, Batzer MA, Gurven M, Jorde LB, Rogers AR, et al (1998) Genetic traces of ancient demography. Anthropology 95: 1961–1967. 10.1073/pnas.95.4.1961 PMC19224 9465125

[18] Kapuscinski AR, Miller LM (2007) Genetic guidelines for fisheries management. Minnesota: Minnesota Sea Grant. 113p.

[19] Mu XD, Hu YC, Wang XJ, Song HM, Yang YX, et al (2011) Genetic variability in cultured stocks of *Scleropages formosus* in mainland China revealed by microsatellite markers. Journal of Animal and Veterinary Advances 10(5): 555–561.

[20] de Bruyn M, Nugroho E, Mokarrom Hossain Md, Wilson JC, Mather PB (2005) Phylogeographic evidence for the existence of an ancient biogeographic barrier: the Isthmus of Kra Seaway. Heredity 94: 370–378. 15523504 10.1038/sj.hdy.6800613

[21] Helgason A, Lalueza-Fox C, Ghosh S, Siguròardóttir S, Sampietro ML, et al.. (2009) Sequences from first settlers reveal rapid evolution in Icelandic mtDNA pool. PLoS Genetics 5(1), e1000343. doi:10.1371/journal.pgen.1000343. 10.1371/journal.pgen.1000343 PMC2613751 19148284

[22] Berezina G, Svyatova G, Makhmutova Z (2011) The analysis of the genetic structure of the Kazakh population as estimated from mitochondrial DNA polymorphism. Medical and Health Science Journal 6: 2–6.

[23] Ballard JW, Whitlock MC (2004) The incomplete natural history of mitochondria. Molecular Ecology 13(4): 729–744. 15012752 10.1046/j.1365-294x.2003.02063.x

[24] Jiggins FM (2003) Male-killing Wolbachia and mitochondrial DNA: selective sweeps, hybrid introgression and parasite population dynamics. Genetics 164(1): 5–12. 12750316 10.1093/genetics/164.1.5 PMC1462540

[25] Bazin E, Glémin S, Galtier N (2006) Population size does not influence mitochondrial genetic diversity in animals. Science 312(5773): 570–572. 16645093 10.1126/science.1122033

[26] Galtier N, Nabholz B, Glémin S, Hurst GDD (2009) Mitochondrial DNA as a marker of molecular diversity: a reappraisal. Molecular Ecology 18: 4541–4550. 19821901 10.1111/j.1365-294X.2009.04380.x

[27] Hurst GDD, Jiggins FM (2005) Problems with mitochondrial DNA as a marker in population, phylogeographic and phylogenetic studies: the effect of inherited symbionts. Proceedings of the Royal Society B 272: 1525–1534. 16048766 10.1098/rspb.2005.3056 PMC1559843

[28] Tamura K, Dudley J, Nei M, Kumar S (2007) MEGA4: Molecular Evolutionary Genetics Analysis (MEGA) software version 4.0. Molecular Biology and Evolution 10.1093/molbev/msm092. 10.1093/molbev/msm092 17488738

[29] Posada D (2004) Collapse ver. 1.2. A tool for collapsing sequences to haplotypes. Available: http://Darwin.uvigo.es. Accessed 2010 September 21.

[30] Rozas J, Sanchez-DelBarrio JC, Messeguer X, Rozas R (2003) DnaSP, DNA polymorphism analyses by the coalescent and other methods. Bioinformatics 19: 2496–2497. 14668244 10.1093/bioinformatics/btg359

[31] Excoffier L, Laval G, Schneider S (2005) Arlequin ver. 3.0: An integrated software package for population genetics data analysis. Evolutionary Bioinformatics Online 1: 47–50. PMC2658868 19325852

[32] Saitou N, Nei M (1987) The neighbor-joining method: a new method for reconstructing phylogenetic trees. Molecular Biology and Evolution 4(4): 406–425. 3447015 10.1093/oxfordjournals.molbev.a040454

[33] Drummond AJ, Rambaut A (2007) BEAST: Bayesian evolutionary analysis by sampling trees. BMC Evolutionary Biology 7: 214. 17996036 10.1186/1471-2148-7-214 PMC2247476

[34] Wang J, Yang G (2011) The complete mitogenome of the snakehead *Channa argus* (Perciformes:Channoidei): genome characterization and phylogenetic implications. Mitochondrial DNA 22(4): 120–129. 22040081 10.3109/19401736.2011.624599

[35] Kimura M (1980) A simple method for estimating evolutionary rate of base substitutions through comparative studies of nucleotide sequences. Journal of Molecular Evolution 16: 111–120. 7463489 10.1007/BF01731581

[36] Felsenstein J (1985) Confidence limits on phylogenies: An approach using the bootstrap. Evolution 39: 783–791. 28561359 10.1111/j.1558-5646.1985.tb00420.x

[37] Drummond AJ, Ho SYW, Phillips MJ, Rambaut A (2006) Relaxed phylogenetics and dating with confidence. PLoS Biology 4(5), e88. doi:10.1371/journal.pbio.0040088. 10.1371/journal.pbio.0040088 PMC1395354 16683862

[38] Kingman JFC (1982) The coalescent. Stochastic Processes and their Application 13: 235–248.

[39] Rambaut A, Drummond AJ (2007) Tracer v1.4. Available: http://tree.bio.ed.ac.uk/software/tracer. Accessed 2012 May 5.

[40] Rambaut A (2009). FigTree v1.3.1. Available: http://tree.bio.ed.ac.uk/software/figtree/. Accessed 2012 May 5.

[41] Bandelt HJ, Forster P, Rohl A (1999) Median-joining networks for inferring intraspecific phylogenies. Molecular Biology and Evolution 16: 37–48. 10331250 10.1093/oxfordjournals.molbev.a026036

[42] Dupanloup, Schneider S, Excoffier L (2002) A simulated annealing approach to define the genetic structure of populations. Molecular Ecology 11: 2571–2581. 12453240 10.1046/j.1365-294x.2002.01650.x

[43] Benjamini Y, Hochberg Y (1995) Controlling the false discovery rate: a practical and powerful approach to multiple testing. Journal of the Royal Statistical Society B 57(1): 289–300.

[44] Lynch M, Crease TJ (1990) The analysis of population survey data on DNA sequence variation. Molecular Biology and Evolution 7(3): 377–394. 1974693 10.1093/oxfordjournals.molbev.a040607

[45] Hudson RR, Boos DD, Kaplan NL (1992) A statistical test for detecting geographic subdivision. Molecular Biology and Evolution 9(1): 138–151. 1552836 10.1093/oxfordjournals.molbev.a040703

[46] Nei M (1973) Analysis of gene diversity in subdivided populations. Proceedings of the National Academy of Sciences of the United States of America 70(12) Part I: 3321–3323. 10.1073/pnas.70.12.3321 PMC427228 4519626

[47] Miya M, Saitoh K, Wood R, Nishida M, Mayden RL (2006) New primers for amplifying and sequencing the mitochondrial ND4/ND5 gene region of the Cypriniformes (Actinopterygii: Ostariophysi). Ichthyological Research 53: 75–81.

[48] Nguyen TTT, Hurwood D, Mather PD, Na-Nakorn U, Kamonrat W, et al (2006a) Manual on application of molecular tools in aquaculture and inland fisheries management, Part I: Conceptual basis of population genetic approaches. Bangkok: NACA Monograph No. 1: 80p.

[49] Bernatchez L, Wilson CC (1998) Comparative phylogeography of Neartic and Paleartic fishes. Molecular Ecology 7(4): 431–452.

[50] Roos H (2004) Genetic diversity in the anabantids *Sandelia capensis* and *S. bainsii*: a phylogeography and phylogenetic investigation. MSc thesis, University of Pretoria.

[51] Adamson EAS (2010) Influence of historical landscapes, drainage evolution and ecologic traits of genetic diversity in Southeast Asian freshwater snakehead fishes. PhD thesis, Queensland University of Technology.

[52] Wang JP, Hsu KC, Chiang TY (2000) Mitochondrial DNA phylogeography of *Acrossocheilus paradoxus* (Cyprinidae) in Taiwan. Molecular Ecology 9(10): 1483–1494. 11050544 10.1046/j.1365-294x.2000.01023.x

[53] Chubb AL, Zink RM (1998) Fitzsimons (1998) Patterns of mtDNA variation in Hawaiian freshwater fishes: the phylogeographic consequences of Amphidromy. Journal of Heredity 89: 8–16. 9487675 10.1093/jhered/89.1.8

[54] Newman D, Pilson D (1997) Increased probability of extinction due to decreased genetic effective population size: experimental populations of *Clarkia pulchella* . Evolution 51(2): 354–362. 28565367 10.1111/j.1558-5646.1997.tb02422.x

[55] Hauser L, Adcock GJ, Smith PJ, Bernal Ramirez JH, Carvalho GR (2002) Loss of microsatellite diversity and low effective population size in an overexploited population of New Zealand snapper (*Pagrus auratus*). Proceedings of the National Academy of Sciences of the United States of America 99(18): 11742–11747. 12185245 10.1073/pnas.172242899 PMC129339

[56] Luikart G, Sherwin WB, Steele BM, Allendorf FW (1998) Usefulness of molecular markers for detecting population bottlenecks via monitoring genetic change. Molecular Ecology 7: 963–974. 9711862 10.1046/j.1365-294x.1998.00414.x

[57] Wang ZW, Zhou JF, Ye YZ, Wei QW, Wu QJ (2006) Genetic structure and low genetic diversity suggesting the necessity for conservation of the Chinese Longsnout Catfish, *Leiocassis longirostris* (Pisces: Bagriidae). Environmental Biology of Fish 75(4): 455–463.

[58] Zwick A, Regier JC, Mitter C, Cummings MP (2011) Increased gene sampling yields robust support for higher-level clades within Bombycoidea (Lepidoptera). Systematic Entomology 36: 31–43.

[59] Zakharov EV, Lobo NF, Nowak C, Hellman JJ (2009) Introgression as likely cause of mtDNA paraphyly in two allopatric skippers (Lepidoptera: Hesperiidae). Heredity 102: 590–599. 19293835 10.1038/hdy.2009.26

[60] Yu L, Li YW, Ryder OA, Zhang YP (2007) Analysis of complete mitochondrial genome sequences increases phylogenetic resolution of bears (Ursidae), a mammalian family that experienced rapid speciation. BMC Evolutionary Biology 7: 198. 17956639 10.1186/1471-2148-7-198 PMC2151078

[61] Dodson JJ, Colombani F, Ng PKL (1995) Phylogeographic structure in mitochondrial DNA of a Southeast Asian freshwater fish, *Hemibagrus nemurus* (Siluroidei; Bagridae) and Pleistocene sea-level changes on the Sunda shelf. Molecular Ecology 4: 331–346.

[62] Inger RF, Chin PK (1962) The Fresh-water Fishes of North Borneo. Fieldiana: Chicago Natural History Museum. 352p.

[63] Ryan JR, Esa YB (2006) Phylogenetic analysis of *Hampala* fishes (subfamily Cyprinidae) in Malaysia inferred from partial mitochondrial cytochrome b DNA sequences. Zoological Sciences 23(10): 893–901. 10.2108/zsj.23.893 17116992

[64] Esa Y, Siraj SS, Daud SK, Rahim KAA, Japning JRR, et al (2008) Mitochondrial DNA diversity of *Tor tambroides* Valenciennes (Cyprinidae) from five natural populations in Malaysia. Zoological Studies 47(3): 360–367.

[65] Leong KO (2003) Classification and genetic variation of the genus *Labiobarbus* (Cyprinidae) in Peninsular Malaysia: reviewed by morphological and molecular techniques. MSc thesis, Universiti Sains Malaysia.

[66] Schuster WH (1952) A provisional survey of the introduction and transplantation of fish throughout the Indo-Pacific region. Third IPFC Fisheries Symposium: 187–196.

[67] Ramstad KM, Woody CA, Sage GK, Allendorf FW (2004) Founding events influence genetic population structure of sockeye salmon (*Oncorhynchus nerka*) in Lake Clark, Alaska. Molecular Ecology 13: 277–290. 14717887 10.1046/j.1365-294x.2003.2062.x

[68] Dominguez-Dominguez O, Alda F, Perez-Ponce de Leon G, Garcia-Garitagoitia JL, Doadrio I (2008) Evolutionary history of the endangered fish *Zoogoneticus quitzeoensis* (Bean, 1898) (Cyprinodontiformes: Goodeidae) using a sequential approach to phylogeography based on mitochondrial and nuclear DNA data. BMC Evolutionary Biology 8: 161. 18503717 10.1186/1471-2148-8-161 PMC2435552

[69] Michel C, Hicks BJ, Stölting KN, Clarke AC, Stevens MI, et al. (2008) Distinct migratory and non-migratory ecotypes of an endemic New Zealand eleotrid (*Gobiomorphus cotidianus*)-implications for incipient speciation in island freshwater fish species. BMC Evolutionary Biology 8(49), doi:10.1186/1471-2148-8-49. 10.1186/1471-2148-8-49 PMC2270262 18275608

