# Correction: Phylogeographic Pattern of the Striped Snakehead, *Channa striata* in Sundaland: Ancient River Connectivity, Geographical and Anthropogenic Singnatures

**DOI:** 10.1371/annotation/2be4a0a9-b3ed-492f-8c4d-15d3a5ae6cab

**Published:** 2013-05-24

**Authors:** Min Pau Tan, Amirul Firdaus Jamaluddin Jamsari, Mohd Nor Siti Azizah

There was an error in the title of the article.

The correct title is: Phylogeographic Pattern of the Striped Snakehead, Channa striata in Sundaland: Ancient River Connectivity, Geographical and Anthropogenic Signatures

The correct Citation is: Tan MP, Jamsari AFJ, Siti Azizah MN (2012) Phylogeographic Pattern of the Striped Snakehead, Channa striata in Sundaland: Ancient River Connectivity, Geographical and Anthropogenic Signatures. PLoS ONE 7(12): e52089. doi:10.1371/journal.pone.0052089 

