# Supplementary material for: Phylogeographic Pattern of the Striped Snakehead, Channa striata in Sundaland: Ancient River Connectivity, Geographical and Anthropogenic Singnatures
Source: PLoS One. 2012 Dec 20;7(12):e52089. doi: 10.1371/journal.pone.0052089 (PMC3527338; doi:10.1371/journal.pone.0052089)
Supplement: Table S1 — Haplotype frequency and nucleotide polymorphic sites encoded by 27 haplotypes. Non-synonymous amino acid substitution is indicated by * (DOCX) [file pone.0052089.s001.docx]

Table S1. Haplotype frequency and nucleotide polymorphic sites encoded by 27 haplotypes.

|  |  |  |  |  |  | 1 | 1 | 2 | 2 | 2 | 2 | 3 | 3 | 3 | 3 | 3 | 3 | 3 | 4 | 4 | 4 | 4 | 4 | 4 | 4 | 5 | 5 | 5 | 5 | 5 | 5 | 5 | 5 | 5 | 6 | 6 | 6 | 6 | 6 | 6 | 6 | 7 | 7 | 7 | 7 | 7 | 8 | 8 | 8 | 8 | 8 | 9 | 9 | Haplotype |
| --- | --- | --- | --- | --- | --- | --- | --- | --- | --- | --- | --- | --- | --- | --- | --- | --- | --- | --- | --- | --- | --- | --- | --- | --- | --- | --- | --- | --- | --- | --- | --- | --- | --- | --- | --- | --- | --- | --- | --- | --- | --- | --- | --- | --- | --- | --- | --- | --- | --- | --- | --- | --- | --- | --- |
|  | 1 | 2 | 8 | 8 | 8 | 5 | 6 | 0 | 1 | 2 | 2 | 0 | 1 | 3 | 6 | 7 | 8 | 9 | 1 | 5 | 5 | 6 | 6 | 7 | 8 | 0 | 1 | 2 | 3 | 3 | 5 | 6 | 7 | 8 | 0 | 3 | 6 | 7 | 8 | 9 | 9 | 2 | 3 | 5 | 5 | 7 | 0 | 2 | 3 | 4 | 5 | 0 | 5 | frequency |
|  | 8 | 1 | 3 | 7 | 8 | 4 | 6 | 0 | 6 | 5 | 8 | 0 | 8 | 0 | 0 | 5 | 7 | 0 | 7 | 0 | 9 | 0 | 2 | 4 | 0 | 1 | 6 | 5 | 1 | 7 | 5 | 4 | 3 | 5 | 8 | 6 | 9 | 2 | 7 | 6 | 9 | 3 | 2 | 0 | 2 | 1 | 2 | 8 | 5 | 9 | 8 | 7 | 4 |  |
| Hap01 | C | T | A | G | C | T | A | G | C | C | C | C | A | A | C | C | T | C | A | G | T | T | G | A | A | T | A | C | A | C | A | A | T | G | C | C | T | G | A | G | G | C | C | A | A | T | C | T | T | T | A | G | T | 0.1607 |
| Hap02 | . | . | . | . | . | . | . | . | A* | . | . | . | . | . | . | . | . | . | . | . | . | . | A | G | . | . | . | . | . | . | . | . | . | . | . | . | . | . | . | A | . | . | . | . | . | . | . | . | . | . | . | . | C | 0.1536 |
| Hap03 | . | . | G* | C | . | . | . | . | . | . | T | . | . | . | . | . | . | . | . | . | . | . | . | G | . | . | . | . | . | . | . | . | . | . | . | . | . | . | . | A | A | T | . | . | . | . | . | . | . | C | . | . | C | 0.1464 |
| Hap04 | . | . | G* | . | . | . | . | . | . | . | T | . | . | . | . | . | . | . | . | . | . | . | . | G | . | . | . | . | . | . | . | . | . | . | . | . | . | . | . | A | A | T | . | . | . | . | . | . | C | C | . | . | C | 0.0929 |
| Hap05 | . | . | . | . | . | . | . | . | . | . | . | . | . | . | . | . | . | . | . | . | C | . | . | . | . | . | . | . | . | . | . | . | . | . | . | . | . | . | . | . | . | . | . | . | . | . | . | . | . | . | . | . | . | 0.0857 |
| Hap06 | . | . | . | . | . | . | . | . | . | . | . | . | . | . | . | . | . | . | G | . | . | . | . | G | . | . | . | . | . | . | . | . | . | . | . | . | . | . | . | A | . | . | . | . | . | . | . | . | . | . | . | . | C | 0.0714 |
| Hap07 | . | . | G* | . | . | . | G* | . | . | . | T | . | . | . | . | . | . | . | . | . | . | . | . | G | . | . | . | . | . | . | . | . | . | . | . | . | . | . | . | A | A | T | . | . | . | . | . | . | . | C | . | . | C | 0.0714 |
| Hap08 | . | . | G* | . | A* | . | . | . | . | . | T | . | . | . | . | . | . | . | . | . | . | . | . | G | . | . | . | . | . | . | . | . | . | . | . | T | . | . | . | A | A | T | . | . | . | . | . | . | . | C | . | . | C | 0.0607 |
| Hap09 | . | . | . | . | . | . | . | . | A* | . | . | . | . | . | . | . | . | T | . | . | . | . | A | G | . | . | G | . | G | . | . | . | . | . | . | . | . | . | . | A | . | . | . | . | . | . | . | . | . | . | . | . | C | 0.025 |
| Hap10 | . | . | . | . | . | . | . | . | A* | . | . | . | . | . | . | . | . | T | . | . | . | . | A | G | . | . | G | . | G | . | G | . | . | . | . | . | . | . | . | A | . | . | . | . | . | . | . | . | . | . | . | . | C | 0.0179 |
| Hap11 | . | . | G* | . | . | . | . | . | . | T | T | . | . | . | . | . | . | . | . | . | . | . | . | G | . | C | . | . | . | . | . | . | . | . | T* | . | . | . | . | A | . | T | . | . | . | . | . | . | . | C | . | . | C | 0.0143 |
| Hap12 | . | . | G* | . | . | . | . | A* | . | . | T | . | . | . | . | . | . | . | . | . | . | . | . | G | . | . | . | . | G | . | . | . | . | . | . | . | . | . | . | A | A | T | . | . | . | . | . | . | . | C | . | . | C | 0.0107 |
| Hap13 | . | G | G* | . | . | . | . | . | . | . | T | . | . | . | . | . | . | . | . | . | . | . | . | G | . | . | . | . | G | . | . | . | . | . | . | . | . | . | . | A | A | T | . | . | . | . | . | . | C | C | . | . | C | 0.0107 |
| Hap14 | . | . | G* | . | . | . | . | . | . | . | T | . | . | . | . | . | . | . | . | . | . | . | . | G | . | . | . | . | G | . | . | . | . | . | . | . | . | . | . | A | A | T | . | . | . | . | . | . | C | C | . | . | C | 0.0107 |
| Hap15 | . | . | G* | A | . | . | . | . | . | T | T | . | . | . | . | . | . | . | . | . | . | . | . | G | . | . | . | . | . | . | . | . | . | . | . | . | . | . | . | A | . | T | . | . | . | . | . | . | . | C | . | . | C | 0.0071 |
| Hap16 | . | . | . | . | . | . | . | A* | A* | . | . | . | . | . | . | . | . | T | . | . | . | . | A | G | . | . | G | . | G | . | . | . | . | . | . | . | . | . | . | A | . | . | . | . | . | . | . | . | . | . | . | . | C | 0.0071 |
| Hap17 | . | . | . | . | . | . | . | . | A* | . | . | . | . | . | . | . | . | T | . | . | . | . | A | G | . | . | G | . | G | . | . | . | . | . | . | . | . | . | . | A | . | . | . | . | . | . | . | . | . | . | . | A* | C | 0.0036 |
| Hap18 | . | . | . | . | . | . | . | A* | A* | . | . | . | . | . | . | A | . | T | . | . | . | . | A | G | . | . | G | . | G | . | . | . | . | . | . | . | . | . | . | A | . | . | . | . | . | . | . | . | . | . | . | . | C | 0.0036 |
| Hap19 | G | . | . | . | . | . | . | . | . | . | . | . | . | . | . | . | . | . | . | . | C | . | . | . | . | C | . | . | . | . | . | . | . | . | . | . | . | . | . | . | . | . | . | . | . | . | . | . | . | . | . | . | . | 0.0036 |
| Hap20 | . | . | G* | . | . | . | . | . | . | T | T | . | G | . | . | . | . | . | . | . | . | . | . | G | . | . | . | . | . | . | . | . | . | A | . | . | . | . | . | A | . | T | . | . | . | . | . | . | . | C | . | . | C | 0.0036 |
| Hap21 | . | . | . | . | . | . | . | . | . | . | . | . | . | . | . | . | . | . | . | . | . | . | . | . | . | . | . | . | . | . | . | . | . | . | . | . | . | . | . | . | . | . | A | . | . | . | . | . | . | . | . | . | . | 0.0036 |
| Hap22 | . | . | G* | . | . | . | G* | . | . | . | T | . | . | . | . | . | . | . | . | . | . | . | . | G | . | . | . | . | . | . | . | . | . | . | . | . | . | . | T | A | A | T | . | . | . | . | . | . | . | C | . | . | C | 0.0036 |
| Hap23 | . | . | . | . | . | . | . | . | . | . | . | . | . | C | . | . | . | . | . | . | . | . | . | . | . | . | . | . | . | . | . | . | . | . | . | . | . | . | . | . | . | . | . | . | . | . | . | . | . | . | . | . | . | 0.0036 |
| Hap24 | . | . | . | . | . | . | . | . | . | . | . | . | . | . | . | . | . | . | . | . | . | . | . | G | . | . | . | . | . | . | . | . | . | . | . | . | . | . | . | A | . | . | . | . | . | . | . | . | . | . | . | . | C | 0.0036 |
| Hap25 | . | . | . | A | . | C* | . | . | . | . | T | T | . | . | T | . | C | . | . | A | . | C | . | G | G | . | . | T | G | T | . | G | C | . | . | . | C | A | . | A | A | . | . | T | G | C | A* | C | . | . | G | . | C | 0.0036 |
| Hap26 | . | . | . | . | . | . | . | . | A* | . | . | . | . | . | . | . | . | T | . | . | . | . | A | G | . | . | G | . | G | . | G | . | . | . | . | . | . | A | . | A | . | . | . | . | . | . | . | . | . | . | . | . | C | 0.0036 |
| Hap27 | . | . | G* | . | . | . | . | . | . | . | T | . | . | . | . | . | . | . | . | . | . | . | . | G | . | . | . | . | . | . | . | . | . | . | . | . | . | . | . | A | A | T | . | . | . | . | . | . | . | C | . | . | C | 0.0179 |

Non-synonymous amino acid substitution is indicated by *
