# Supplementary material for: Phylogeographic Pattern of the Striped Snakehead, Channa striata in Sundaland: Ancient River Connectivity, Geographical and Anthropogenic Singnatures
Source: PLoS One. 2012 Dec 20;7(12):e52089. doi: 10.1371/journal.pone.0052089 (PMC3527338; doi:10.1371/journal.pone.0052089)
Supplement: Table S2 — Haplotype distribution across 24 populations. Abbreviation of population name is as listed in Table 1. (DOCX) [file pone.0052089.s002.docx]

**Table S2. Haplotype distribution across 24 populations.**

|  | Northwest Peninsular | | | | | | Central west Peninsular | | | Southern Peninsular | | | East Peninsular | | | | | | Malaysian Borneo | | | | Sumatra | |  |
| --- | --- | --- | --- | --- | --- | --- | --- | --- | --- | --- | --- | --- | --- | --- | --- | --- | --- | --- | --- | --- | --- | --- | --- | --- | --- |
|  | TT | KN | JN | TK | SP | KR | TR | TP | KJ | LG | YP | MS | BJ | KB | TL | SG | KT | KK | SW | SS | SB | KS | CS | KP | Total |
| Hap01 |  |  | 4 | 15 | 12 | 13 | 1 |  |  |  |  |  |  |  |  |  |  |  |  |  |  |  |  |  | 45 |
| Hap02 |  |  |  |  |  | 1 |  |  | 4 |  |  |  |  |  |  |  |  | 1 |  | 10 | 14 | 13 |  |  | 43 |
| Hap03 |  |  |  |  |  |  |  |  |  |  |  |  | 10 | 15 |  |  | 16 |  |  |  |  |  |  |  | 41 |
| Hap04 |  |  |  |  |  |  |  |  |  | 4 | 10 | 7 |  |  | 5 |  |  |  |  |  |  |  |  |  | 26 |
| Hap05 | 6 | 13 |  |  |  |  | 1 |  |  |  |  |  |  |  |  | 3 |  | 1 |  |  |  |  |  |  | 24 |
| Hap06 |  |  | 1 |  |  |  | 7 | 10 |  |  |  |  |  |  |  |  |  | 2 |  |  |  |  |  |  | 20 |
| Hap07 |  |  |  |  |  |  |  |  |  | 1 |  | 6 |  |  | 2 | 2 |  | 9 |  |  |  |  |  |  | 20 |
| Hap08 |  |  |  |  |  |  |  |  |  |  |  |  |  |  |  |  |  |  | 15 | 2 |  |  |  |  | 17 |
| Hap09 |  |  |  |  |  |  | 2 |  | 4 |  |  |  |  |  |  |  |  |  |  |  |  | 1 |  |  | 7 |
| Hap10 |  |  |  |  |  |  | 4 |  | 1 |  |  |  |  |  |  |  |  |  |  |  |  |  |  |  | 5 |
| Hap11 |  |  |  |  |  |  | 2 |  | 2 |  |  |  |  |  |  |  |  |  |  |  |  |  |  |  | 4 |
| Hap12 |  |  |  |  |  |  |  |  |  |  |  |  |  | 1 |  | 2 |  |  |  |  |  |  |  |  | 3 |
| Hap13 |  |  |  |  |  |  |  |  |  |  |  |  |  |  |  |  |  |  |  |  |  |  | 3 |  | 3 |
| Hap14 |  |  |  |  |  |  |  |  |  |  |  |  |  |  |  |  |  |  |  |  |  |  | 3 |  | 3 |
| Hap15 |  |  |  |  |  |  |  |  | 2 |  |  |  |  |  |  |  |  |  |  |  |  |  |  |  | 2 |
| Hap16 |  |  |  |  |  | 1 | 1 |  |  |  |  |  |  |  |  |  |  |  |  |  |  |  |  |  | 2 |
| Hap17 |  |  | 1 |  |  |  |  |  |  |  |  |  |  |  |  |  |  |  |  |  |  |  |  |  | 1 |
| Hap18 |  |  |  |  |  |  |  |  | 1 |  |  |  |  |  |  |  |  |  |  |  |  |  |  |  | 1 |
| Hap19 |  | 1 |  |  |  |  |  |  |  |  |  |  |  |  |  |  |  |  |  |  |  |  |  |  | 1 |
| Hap20 |  |  |  |  |  |  |  |  |  |  |  |  |  | 1 |  |  |  |  |  |  |  |  |  |  | 1 |
| Hap21 |  |  |  |  |  | 1 |  |  |  |  |  |  |  |  |  |  |  |  |  |  |  |  |  |  | 1 |
| Hap22 |  |  |  |  |  |  |  |  |  |  |  |  |  |  |  |  |  | 1 |  |  |  |  |  |  | 1 |
| Hap23 |  |  |  |  | 1 |  |  |  |  |  |  |  |  |  |  |  |  |  |  |  |  |  |  |  | 1 |
| Hap24 |  |  |  |  |  |  |  |  |  | 1 |  |  |  |  |  |  |  |  |  |  |  |  |  |  | 1 |
| Hap25 |  |  |  |  |  |  |  |  |  | 1 |  |  |  |  |  |  |  |  |  |  |  |  |  |  | 1 |
| Hap26 |  |  |  |  |  |  | 1 |  |  |  |  |  |  |  |  |  |  |  |  |  |  |  |  |  | 1 |
| Hap27 |  |  |  |  |  |  |  |  |  |  |  |  |  |  |  |  |  |  |  |  |  |  |  | 5 | 5 |
| Total | 6 | 14 | 6 | 15 | 13 | 16 | 19 | 10 | 14 | 7 | 10 | 13 | 10 | 17 | 7 | 7 | 16 | 14 | 15 | 12 | 14 | 14 | 6 | 5 | 280 |

Populations: TT=Timah Tasoh; KN=Kuala Nerang; JN=Jeniang; TK=Teluk Kumbar; SP=Seberang Prai; KR=Kerian; TR=Tanjung Rambutan; TP=Tapah; KJ=Kajang; LG=Linggi; YP=Yong Peng; MS=Mersing; BJ=Binjai; KB=Kota Bahru; TL=Tanjung Lumpur; SG=Sega; KT=Kubang Bujuk; KK=Kuala Krau; SW=Serian; SS=Sungai Sibuti; SB=Kota Belud; KS=Kampung Kesapang; CS=Takengon; KP=Kampar
